# How Openness Serves Innovation in Healthcare? Comment on "What Managers Find Important for Implementation of Innovations in the Healthcare Sector – Practice Through Six Management Perspectives"

**DOI:** 10.34172/ijhpm.2022.7517

**Published:** 2022-11-14

**Authors:** Élizabeth Côté-Boileau

**Affiliations:** Department of Health Management, Evaluation and Policy, School of Public Health, University of Montreal, Montreal, QC, Canada.

**Keywords:** Open Innovation, Open Strategy, Inclusion, Transparency, Healthcare Management

## Abstract

The recent study of which enabling factors can facilitate the specific step of moving from idea generation to implementation in healthcare supports that managing innovation is a context-driven process that goes through six categories of change. While this research provides a general and rather comprehensives overview of what successful innovation work needs, it does not offer deeper insights into how categories of change can be operated in the context of accelerated openness in healthcare. I use the concepts of open innovation and open strategy to trying better understand how openness, in terms of greater inclusion and transparency, may or may not serve healthcare innovation through three theoretical questions: to whom, how and when to open up to foster innovation? Whilst diversity of knowledge, actors and systems are growing drivers of innovation, strategizing openness for more deliberate and impactful inclusion and transparency in healthcare management is key to coproducing better health.

## Introduction

 Palm and Persson Fischier provide a relevant qualitative analysis of which enabling factors can facilitate the specific step of moving from idea generation to implementation in healthcare. They use an action research methodology to empirically test six complementary theoretical perspectives on innovation and change management with 54 healthcare practitioners involved in a collaborative innovation implementation project in Sweden. The authors found 35 enabling factors that can be regrouped into six categories of change.^1^

 The study reveals that translating healthcare innovation from idea to practice is profoundly context-driven, as different organizations and systems influence the social, institutional, political, policy and economic conditions in which innovations are implemented.^1^ Management is particularly important in creating such a favourable context (operability), and perhaps less in operationalizing the implementation process per se (eg, negotiating the boundaries and possible conflicts that can either facilitate or inhibit innovation circuits).^2^

 In line with Palm and Persson Fischier’s work,^1^ the importance of teamwork, intersectoral collaboration and inclusion are rarely disputed as critical success factors to innovate in healthcare. The plurality and heterogeneity of actors opens up the access to a wider range of knowledge sources, resources and capacities to think and act differently.^3^ Yet, there seems to be a paradoxical tension emerging from the literature, with, on the one hand, the growing interest and need for greater inclusion (range and diversity of actors) and transparency (information sharing) in generating and implementing new ideas, and on the other hand, the generalized difficulty to reconcile competing interests, logics and practices between multiple stakeholders, organizations and systems to stimulate innovation. This leads me to raise the following question: to what extent does openness, in terms of greater inclusion and transparency, serves innovation in healthcare?

 In the following, I will start by briefly looking back at openness as a theoretical concept, and then build on Palm and Persson Fischier’s work^1^ to ask three key questions: to whom, how and when should one open up to foster innovation in healthcare?

## Openness

 Openness originated in the field of innovation as ‘*a paradigm that assumes that firms can and should use external ideas as well as internal ideas, and internal and external paths to market, as they look to advance their technology*.’^4^ In practice, open innovation means increasing the number/range and diversity of actors, resources and knowledge sources that can help solve a given problem. Longstanding research on open innovation generally supports that this practice is beneficial, namely economically, to organizations that leverage knowledge sources and resources from the outside world to improve and innovate internally.^5^

 Open strategy later emerged as a theoretical concept whilst traditional dominant organizational forms (eg, bureaucracies, hierarchies) were challenged and transformed by the rise of “*managerial egalitarianism and mobility; a cultural popularization of strategy; and new technologies that set information free*.”^6^ In healthcare, some scholars characterize this shift as ‘a decentralized narrative’ that triggered new organizational forms (eg, networks) and strategic practices: “*where the State has a diminished role in hands-on service organisations, and instead a multitude of policy actors and stakeholders interact through polycentric networks to coordinate and organise services”*^7^ (p. 3).The two main foundational premises of strategy – that of “exclusion” (strategy as the sole propriety of top management), and that of “secrecy” (information and inimitability as competitive resources) – were progressively disrupted and replaced by two new propositions – that of “inclusion” (strategy gains from a wider scope (range/number) of actors and decision-making rights/depth of participation) and that of “transparency” (strategy gains from a wider scope (range/number) of audiences, topics and degrees of intentionality to share and quality of the information shared). In practice, open strategy is about harnessing collective creativity in making strategy. A growing body of evidence supports that opening strategy increases the legitimacy and commitment of organizational actors into more robust strategic work, and accelerates transformational and innovative work. It is now generally accepted that open innovation is a ‘subtype’ of open strategy.

 I find that openness, in terms of inclusion and transparency, emerge as a transversal component of the six categories of change to implement healthcare innovation proposed by the authors.^1^ Yet, the authors remain quite elusive on three important points: to whom, how and when to open up to foster healthcare innovation? In the following, I will attempt to bring some partial answers to these questions, and warmly invite the authors to respond.

###  To Whom to Open up to Foster Innovation in Healthcare?

 Palm and Persson Fischier^1^ stress the importance of networking, connecting and diversifying within and outside one’s organizations to enhance the innovation process. Both intra- and extra-mural connections are needed to diffuse and promote the innovation, as well as to reach a wider scope of resources and sources of knowledge to successfully generate and implement new ideas. However, the authors do not explicitly bring concrete answers to three important questions: (1) How to strategically organize these collaborations in order to make the most of complementary resources and sources of knowledge? (2) How to balance the involvement and diversity of “traditional” (eg, providers, decision-makers, funders, etc) and “non-traditional” (eg, patients, community members, citizens, etc) actors in strategizing innovation? and (3) How to open up to external partners while remaining competitive towards resources availability?

 Whilst knowledge sources are multiplying at an accelerated pace and resources are becoming scarcer, it is increasingly difficult for managers to base their decisions on ‘the right’ source of knowledge. For instance, if an organization wants to value the lived-experience of care as the primary source of knowledge in their innovation strategy, patient participation must be incentivized as a priority. In a different scenario, an organization could include a hospital CEO as an ‘advisory’ partner with the capacity to provide feedback but without any decision-making rights to remain competitive. This namely highlights how continuums of inclusion and transparency can quickly become issues of shared governance and accountability in innovating in a healthcare context.

 We must consider more seriously the impact of those we include and how openly we include them, as it affects the configurations and boundaries through which knowledge flows interact and on which decisions and policies are made.^8^

###  How to Open up to Foster Innovation in Healthcare?

 Palm and Persson Fischier^1^ call for the importance of the ‘tacit’ work, particularly in the form of discourse, accomplished by managers to enable innovation: “*it is important that managers do not talk about innovation, but about what generative images one has of the future*.”^1^ This work must be inclusive and transparent to increase the chances of success of the ideas generated in terms of perceived value and feasibility by various stakeholders. Openness would both support and emerge from a form of ‘open’ strategic learning (generating actionable knowledge in support of future strategic initiatives and organizational performance) to foster innovation.

 The authors also highlight the need to generate and nourish an organizational culture that is supportive of innovation (eg, testing change, taking risks) and where innovation is considered a key enabler of organizational performance. Such culture which will encourage, not only now, but also in the future, continued innovation cycles. However, in the context of ‘open’ (multi-centric, interorganizational) innovation work, it can be particularly challenging to find alignment between cultural and structural conditions (eg, governance arrangements) to support innovation. As argued by the authors: “*different organizations create different conditions for concretizing general implementation theories.*”^1^ It is important to put in place explicit mechanisms that will help create consistency and cohesiveness within and across organizations (eg, shared incentives, performance indicators, accountability, etc) to promote innovation work towards, yet beyond, intraorganizational performance. Openness would also support and emerge from a form of ‘open’ capacity building to help organizations adapt to one another and their changing environment to collectively and continuously foster innovation.

 Opening up to foster innovation in healthcare, through inclusive and transparent strategic learning and capacity building, however takes time. It can be challenging for managers to incentivize and maintain innovation work, while the impact on organizational performance is ‘slow’ to be demonstrated. One way to help organizations open up effectively in their innovation process is to deliberately display different levels of inclusion and transparency through time.

###  When to Open up to Foster Innovation in Healthcare?

 Palm and Persson Fischier^1^ temporally frame the innovation process in two sequential phases: the idea generation phase and the implementation phase. Literature on open innovation and strategy generally supports that it is more beneficial to include stakeholders ‘selectively’ and ‘deliberately’ throughout innovation phases, rather than consistently and without a particular focus or intent across the whole process.^5^ In healthcare, both the timing of engagement as well as the consideration of individual or group characteristics can affect the levels of efficiency, equity and ethicality of stakeholder engagement and innovation work. For instance, taking into consideration various levels of health and digital literacy early in engaging people with lived-experience of care promotes the success of innovations. Inversely, engaging patients ‘passively’ in all steps of the innovation process can dilute their role and potential for meaningful contribution, and ultimately lead to token engagement. In Palm and Persson Fischier’s study,^1^ we can appreciate how engaging stakeholders, including patients, into innovation work can be achieved daily through new work routines and information flows across system levels and partners.

 Nevertheless, strategizing ‘when’ to openly engage people in the innovation process, particularly people with lived experience of care, remains a challenge for health system practitioners and managers. According to Usher and Denis^9^: “*characteristics of engagement efforts are consequential … and a deeper understanding of what patient engagement means is needed to develop knowledge useful for innovation in clinical practice and health policy*” (p. 2683-2688).

## Policy Implications

 Asking the question of ‘how openness serves innovation in healthcare’ also has implications for the broader making of public health and social policies.^10^ First, it speaks to the need and importance of developing specific instruments and capacities to co-produce public policies based on evidence and with citizens to appreciate the social and cultural acceptability of health policy and planning.^11^ For instance, it is now generally accepted that effective and innovative home care policies relies on the creation of new patterns of relationships between citizens and public and civil society actors (eg, income support, family caregiver support, community organisations, social economy enterprises, etc) to work closer and in more inclusive and coordinated ways. Second, increasing the range and diversity of knowledgeable actors in making health policies also acknowledges a greater and more strategic agency of non-traditional policy actors (eg, citizens, patients, caregivers) against centralized public policy-making.^12^ As policy networks in welfare states are increasingly complex, diffused and multi-centric, it opens up more room for others to influence knowledge flows and decision-making rules at various levels of government.^13^ Finally, promoting openness and innovation through public policies in healthcare (and vice versa) invite us to adopt a more agentic (future-centric) and less deterministic (structurally-focussed) view of healthcare governance, emphasizing the power, capacity and social accountability of actors within and outside the healthcare realm to contest, transform and create policy narratives that promote more equitable population health and policy outcomes.^7,14^

## Conclusion

 Innovation in the sense of the successful exploitation of new ideas is a founding pillar of health system performance and continuous improvement. Palm and Persson Fischier^1^ emphasize the importance of management in supporting and facilitating the success conditions for an ‘innovation-friendly’ environment. However, in a ‘pandemic’ world where public and policy expectations for greater inclusion, diversity, equity, transparency and science in the operation of health systems have never been higher, perhaps we underestimate the magnitude and complexity of the task accomplished by managers to modify how to innovate responsibly in healthcare. In this commentary, I attempted to explore possible ways to support management practice towards strategically opening up the innovation process through diverse and purposeful knowledge flows, strategic learning, capacity building and continuum-based inclusion and transparency activities. My hope is to stimulate the recognition and further study of the role of managers as catalysts for strategic openness in healthcare, with a particular interest in public policy and governance innovations for more equitable and sustainable population health.^15^

## Ethical issues

 Not applicable.

## Competing interests

 Author declares that she has no competing interests.

## Author’s contribution

 ÉCB is the single author of the paper.
